# Exploring the frontiers: tumor immune microenvironment and immunotherapy in head and neck squamous cell carcinoma

**DOI:** 10.1007/s12672-024-00870-z

**Published:** 2024-01-31

**Authors:** Shaokun Liu, Ru Wang, Jugao Fang

**Affiliations:** Department of Otorhinolaryngology Head and Neck Surgery, Beijing Tongren Hospital, Capital Medical University, Beijing, China

**Keywords:** Head and neck squamous cell carcinoma, Tumor microenvironment, Immunotherapy

## Abstract

The global prevalence of head and neck malignancies positions them as the sixth most common form of cancer, with the head and neck squamous cell carcinoma (HNSCC) representing the predominant histological subtype. Despite advancements in multidisciplinary approaches and molecular targeted therapies, the therapeutic outcomes for HNSCC have only marginally improved, particularly in cases of recurrent or metastatic HNSCC (R/MHNSCC). This situation underscores the critical necessity for the development of innovative therapeutic strategies. Such strategies are essential not only to enhance the efficacy of HNSCC treatment but also to minimize the incidence of associated complications, thus improving overall patient prognosis. Cancer immunotherapy represents a cutting-edge cancer treatment that leverages the immune system for targeting and destroying cancer cells. It's applied to multiple cancers, including melanoma and lung cancer, offering precision, adaptability, and the potential for long-lasting remission through immune memory. It is observed that while HNSCC patients responsive to immunotherapy often experience prolonged therapeutic benefits, only a limited subset demonstrates such responsiveness. Additionally, significant clinical challenges remain, including the development of resistance to immunotherapy. The biological characteristics, dynamic inhibitory changes, and heterogeneity of the tumor microenvironment (TME) in HNSCC play critical roles in its pathogenesis, immune evasion, and therapeutic resistance. This review aims to elucidate the functions and mechanisms of anti-tumor immune cells and extracellular components within the HNSCC TME. It also introduces several immunosuppressive agents commonly utilized in HNSCC immunotherapy, examines factors influencing the effectiveness of these treatments, and provides a comprehensive summary of immunotherapeutic strategies relevant to HNSCC.

## Introduction

HNSCC represents the predominant malignancy in the head and neck region, accounting for approximately 90% of all head and neck cancers and 16–40% of systemic malignancies [1]. Annually, it contributes to 600,000 new cases globally [1]. The incidence and mortality rates of HNSCC have been increasing steadily over the years. Notably, HNSCC is characterized by its heterogeneity, with over 60% of patients presenting with advanced or metastatic disease at diagnosis. Despite the employment of aggressive treatment modalities, including surgery, chemoradiotherapy, or a combination of these approaches, the 5 year overall survival rate for HNSCC, particularly those associated with carcinogens, remains limited to only 40–50% [2, 3]. In addition, conventional therapies may lead to complications. Patients may suffer from functional disabilities or cosmetic defects after surgery, and recurrence remains a huge challenge after incomplete surgical resection. Chemoradiotherapy has systemic toxicity which can damage other organs and also has a risk of pharyngeal dysfunction [4]. Consequently, in cases of R/MHNSCC, the therapeutic options are limited, and the prognosis is generally poor.

T cell-based immunotherapies, including immune checkpoint inhibitors (ICIs), have demonstrated efficacy in increasing the overall survival (OS) rate in patients with R/MHNSCC. ICIs function by reactivating cytotoxic T lymphocytes (CTLs) and are dependent on their ability to target and eliminate tumor cells [4, 5]. While these therapies yield a high rate of sustained response, only a small percentage of HNSCC patients exhibit a favorable response, and challenges such as resistance to immunotherapy remain prevalent. TME plays a crucial role in the pathogenesis, progression, metastasis, diagnosis, and treatment of HNSCC [6]. The TME undergoes dynamic changes that collectively weaken the immune response against cancer. This includes the generation of immunosuppressive factors by tumor and stromal cells, an increase in immune-suppressive cells like Tregs and MDSCs, and the remodeling of the extracellular matrix creating physical barriers. Additionally, metabolic competition and hypoxic conditions further impair immune cell function, while the expression of immune checkpoint molecules like PD-L1 by tumor cells actively inhibits immune attacks. These complex interactions in the TME challenge the effectiveness of the body's natural immune response, underscoring the importance of targeted cancer therapies [5, 6]. Thus, investigating the roles and mechanisms of both anti-tumor and pro-tumor immune cells, as well as extracellular components within the TME of HNSCC, and exploring the significance of tumor immunotherapy in its treatment, can offer new strategies for personalized precision immunotherapy in HNSCC.

## Development of antitumor immune responses

**Fig. 1:**
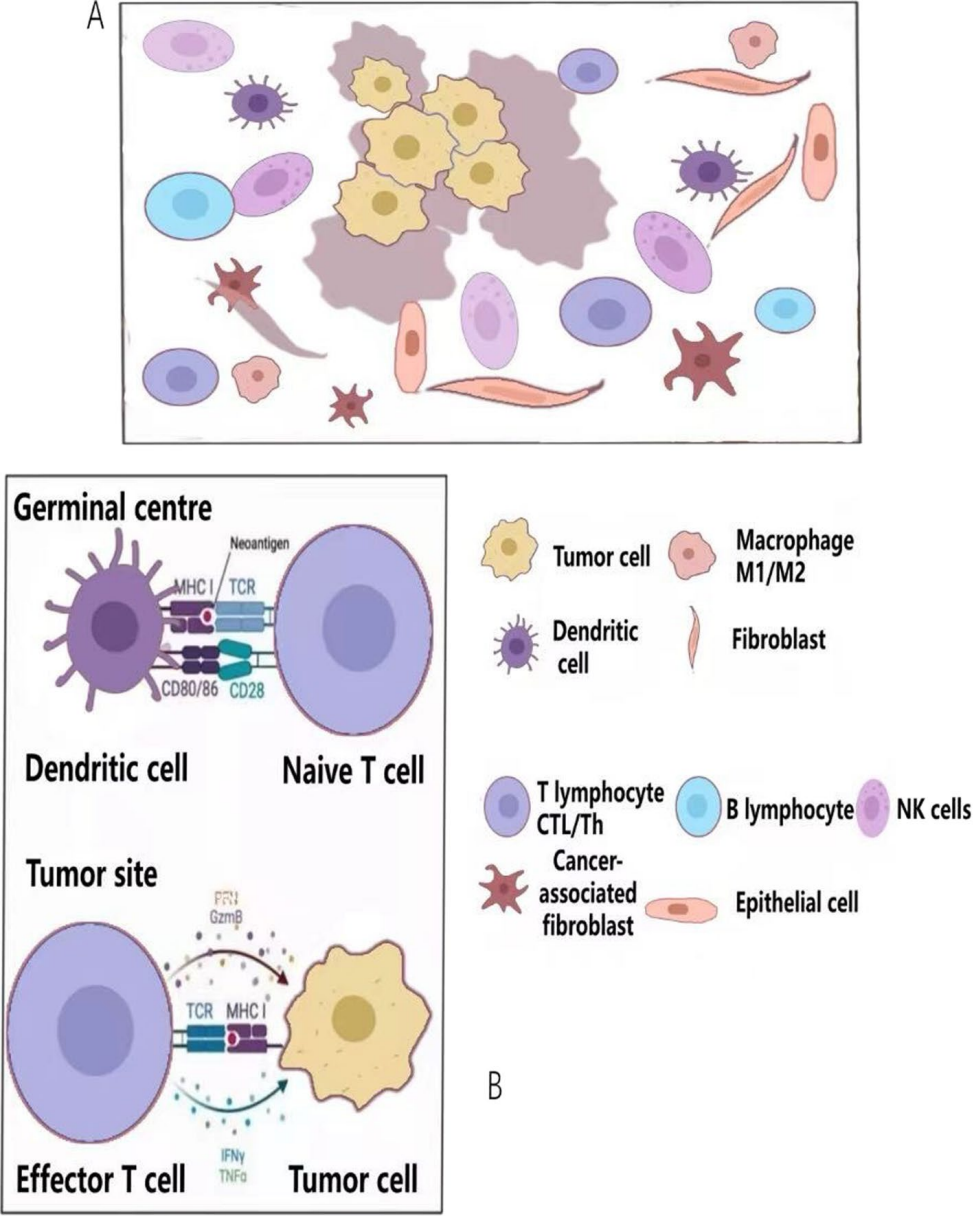
The immune microenvironment of tumors primarily consists of various types of immune-related cells and their interactions. **A**. Common types of immune-related cells present in the tumor's immune microenvironment. **B**. The interactions among these cells constitute the anti-tumor immune activity in the immune microenvironment. *CTL* cytotoxic T lymphocyte, *Th* T helper cell, *NK cells* natural killer cells, *MHC* major histocompatibility complex, *TCR* T cell receptor, *TNF* tumor necrosis factor, *IFN* interferon, *PFN* perforin

TME can elicit dynamic antitumor responses during immune surveillance, as depicted in Fig. 1. T cells possess the capability to recognize tumor cells during the presentation of Tumor-Associated Antigens (TAAs). Originating from mutated genes, TAAs are unique antigenic substances produced by tumor cells, presenting distinct antigenic epitopes (neoantigens) that can effectively stimulate antigen-specific antitumor immune responses [8]. HNSCC is often characterized by a high frequency of p53 tumor suppressor gene loss, which facilitates the formation of TAAs and contributes to genomic instability, thereby enhancing immunogenicity. TAAs, once released by tumor cells, are captured by APCs, such as DCs [9]. These captured TAAs are then presented to the T cell receptor (TCR) via major histocompatibility complex class I (MHC-I) molecules, leading to the activation of CTLs and their migration to tumor tissues. CTLs infiltrate the tumor, attacking and destroying tumor cells through the granular exocytosis of perforin and granzyme [7–9].

**Fig. 2:**
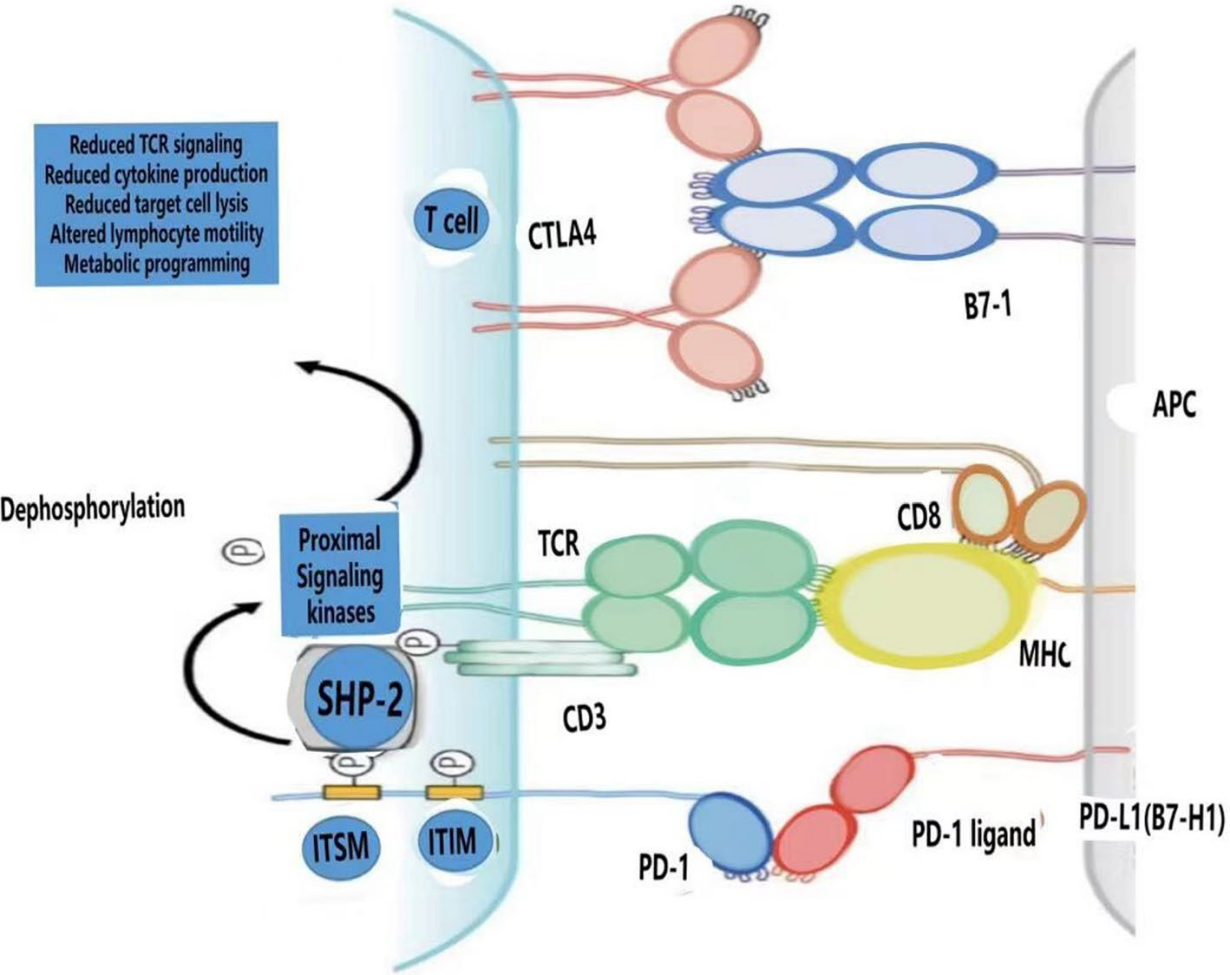
The interaction between PD-1 and PD-L1 leads to a suppression of T lymphocyte function. T cells engage in an interaction with antigen-presenting cells (APCs) through the binding of their surface T cell receptor (TCR) with major histocompatibility complex (MHC) molecules expressed on APCs. The binding of programmed cell death protein 1 (PD-1) with programmed death ligand 1 (PD-L1) leads to an inhibitory signaling pathway. This occurs through the interaction between the immunoreceptor tyrosine-based inhibitory motif (ITIM) of PD-1 and the SH2 domain-containing protein tyrosine phosphatase 2 (SHP-2), resulting in the attenuation of TCR signaling. The diagram also depicts the interaction between another immune checkpoint, cytotoxic T-lymphocyte-associated protein 4 (CTLA-4), and the molecule B7-1. This interaction also transmits a negative regulatory signal, further modulating the activity of T cells

Co-stimulatory molecules, such as B7-1/B7-2 ligands on APCs and CD28 receptors on T cells, play a pivotal role in enhancing T cell activation. Conversely, inhibitory immune checkpoints, including programmed death receptor-1 (PD-1) and cytotoxic T-lymphocyte-associated protein-4 (CTLA-4), which are co-expressed on T cells, act to inhibit T cell activation, thereby contributing to an immunosuppressive response [10, 11]. CTLA-4 competes with CD28 for binding to B7-1 and B7-2 ligands, exhibiting a higher affinity and thus transmitting inhibitory signals to T cells. PD-1 binds to its ligands PD-L1 and PD-L2, leading to the inhibition of T cell activation and promotion of T cell apoptosis [11], as illustrated in Fig. 2.

Certain tumor cells have evolved mechanisms to disrupt, suppress, or evade the immune system by interfering with the normal functions of immune cells. In HNSCC, these mechanisms of immune escape include the generation of new mutant antigens, disruption of antigen presentation pathways, overexpression of PD-L1 inhibitory molecules on CTLs, recruitment of inflammatory cytokines and immunosuppressive agents such as TGF-β, IL-10, IDO, and the presence of key immunosuppressive cells within the TME, including Treg cells and MDSCs [12, 13].

## The role of TME dynamic changes and inhibitory characteristics in HNSCC

**Fig. 3:**
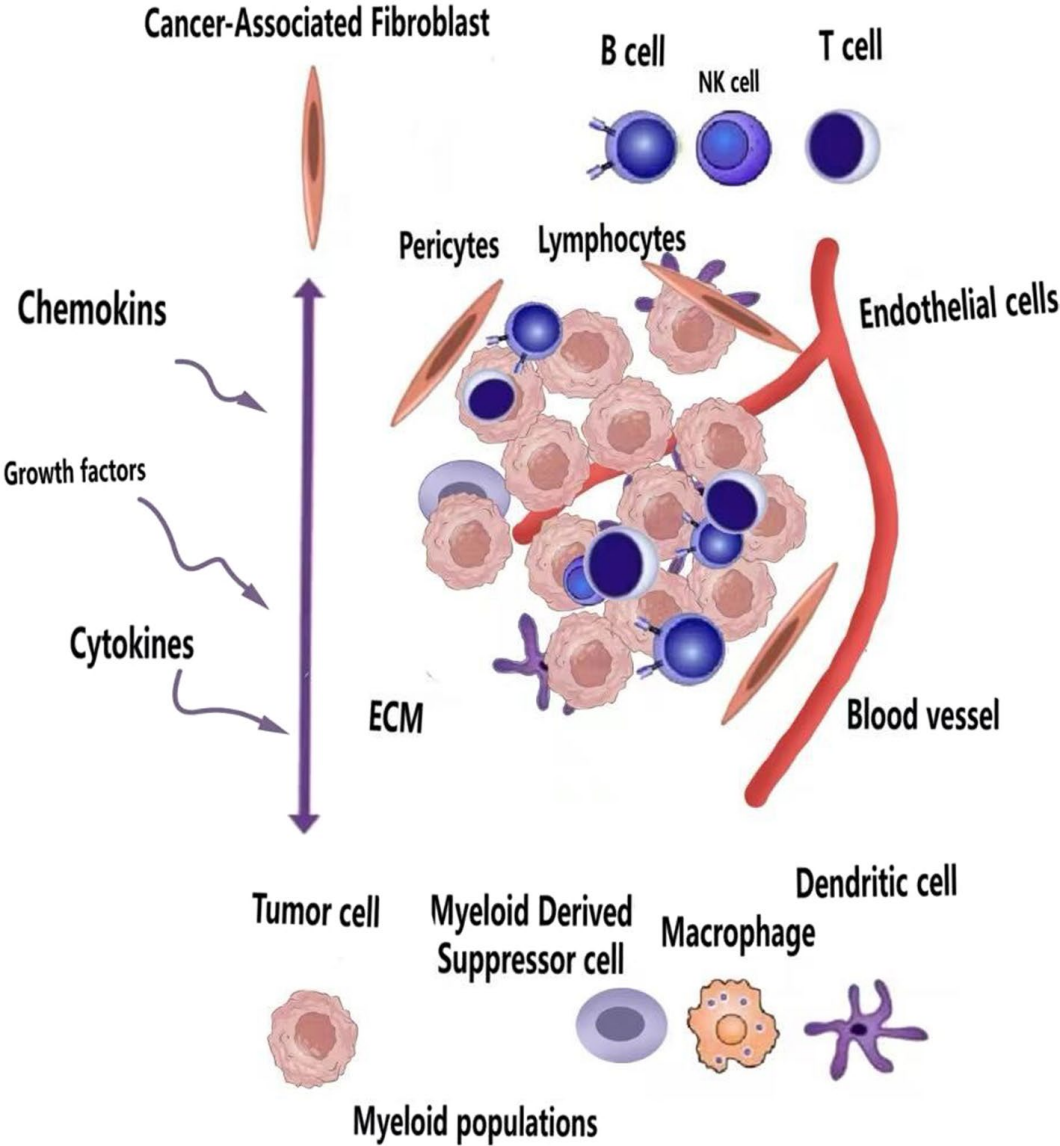
TME encompasses various components, including stromal cells (cancer-associated fibroblasts, endothelial cells, and pericytes), extracellular matrix (ECM), immune cells, and inflammatory cells (T, B, and NK lymphocytes, DCs, macrophages, and myeloid-derived suppressor cells)

TME plays a pivotal regulatory role in the initiation and progression of tumors, encompassing immune and non-immune cells, as well as extracellular components. The immune cell population within the TME includes MDSCs, Treg cells, TAMs, natural killer (NK) cells, and DCs. Non-immune cells are primarily composed of CAFs. Extracellular components include cytokines, growth factors, and ECM [13], as depicted in Fig. 3. These components collectively underscore the significance of the TME in the development and progression of HNSCC. While the body’s immune system is capable of recognizing and eliminating tumor cells, HNSCC can manipulate immune cells within the TME to foster immunosuppression and facilitate immune escape [14]. Research indicates that downregulating human leukocyte antigen (HLA) expression not only aids in immune evasion but also diminishes T cell recognition of tumor cells [13, 14]. Moreover, the TME in HNSCC hosts a subset of cells that inhibit tumor-infiltrating lymphocytes and NK cells, significantly contributing to tumor growth and metastasis [15].

### Immunosuppressive cell

#### Myeloid-derived suppressor cells

**Fig. 4:**
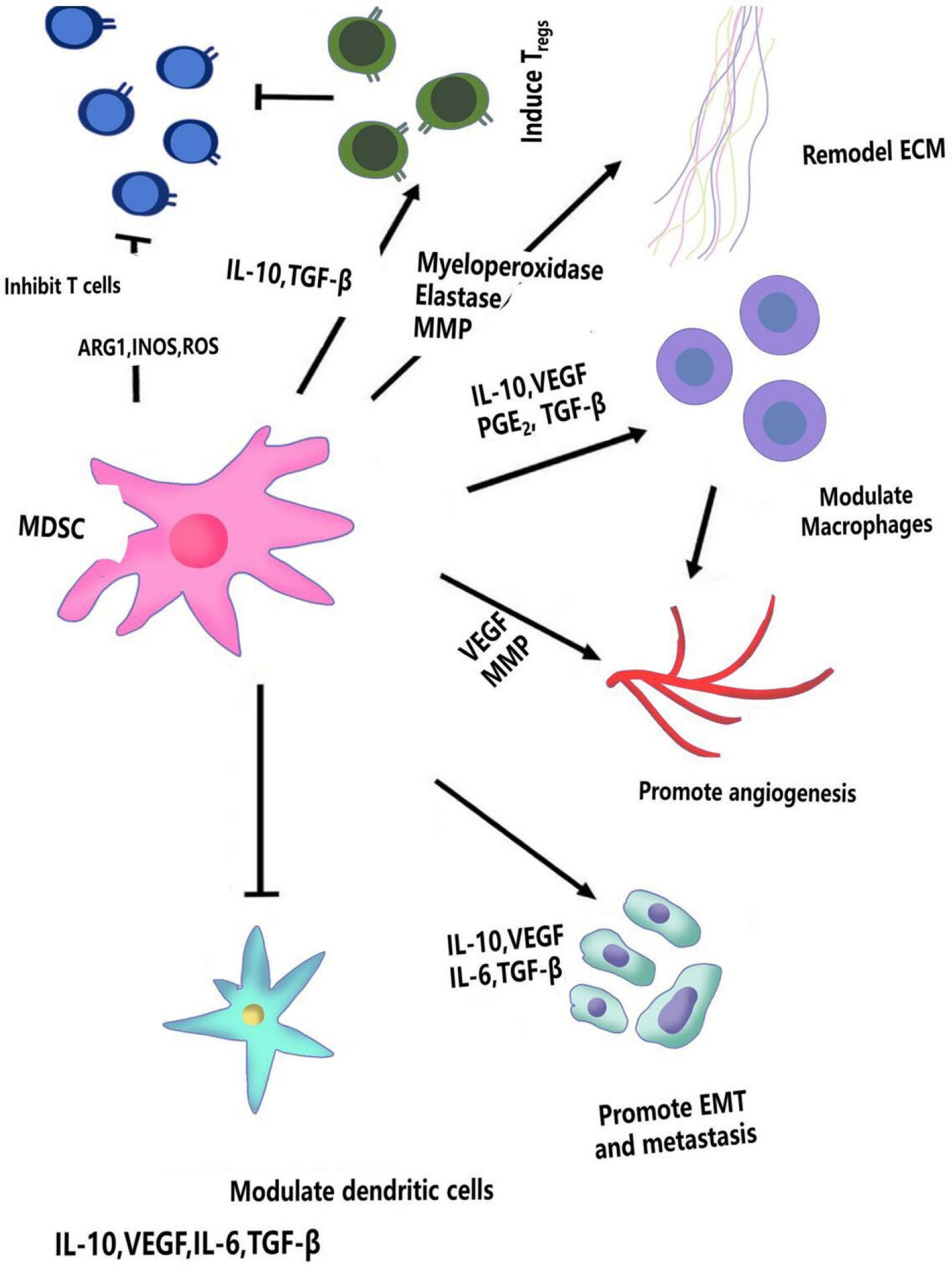
MDSCs contribute multifaceted roles in tumor progression. MDSCs suppress T-cell responses via the release of inhibitory factors, such as arginase 1 (ARG1), inducible nitric oxide synthase (iNOS), and reactive oxygen species (ROS). They facilitate the induction of Tregs and remodel the TME through the secretion of molecules like interleukin-10 (IL-10), transforming growth factor-beta (TGF-β), and vascular endothelial growth factor (VEGF), thereby promoting angiogenesis and metastatic dissemination

MDSCs represent a highly heterogeneous group of immature myeloid cells involved in immune responses, tissue regeneration, tumor metastasis, and angiogenesis through various pathways, as illustrated in Fig. 4. In the TME, MDSCs can inhibit both innate and adaptive immunity through multiple mechanisms, thereby aiding tumor cells in evading immune surveillance and promoting tumor progression [16]. The accumulation of MDSCs in the TME is closely linked to tumor development, poor prognosis, and reduced effectiveness of immunotherapies [17]. During tumorigenesis, MDSCs proliferate rapidly in the TME and exhibit immunosuppressive actions by promoting neovascularization, inhibiting CTLs function, disrupting antigen presentation, differentiating into tumor-associated macrophages (TAMs), and altering natural killer (NK) cell function [18]. Research has demonstrated that MDSCs not only inhibit activated T cells and produce reactive oxygen species, but also interact with T cells to catalyze the nitration of T cell receptors, inducing T cell tolerance [16–18]. In the TME, factors such as vascular endothelial growth factor (VEGF) and IL-6 have been shown to induce MDSC aggregation. In HNSCC, an increase in MDSCs can upregulate inflammatory mediators like IL-1 and IL-6, creating an environment unfavorable for the maturation of APCs, thereby indirectly promoting tumor cell proliferation [18]. Furthermore, MDSCs are known to induce the expansion of Treg cells, with further studies confirming the involvement of IFN-γ, IL-10, and TGF-βin this process.

#### Regulatory T (Treg) cells

Treg cells, a subgroup of inhibitory T cells, express several surface proteins including CTLA-4, glucocorticoid-induced tumor necrosis factor (TNF) receptor, OX40, and CD25. They mediate immunosuppression through various mechanisms [18, 19]. The first mechanism involves the overexpression of IL-2 by Treg cells, leading to the production of inhibitory cytokines such as TGF-β, IL-10, IL-35, and cytotoxic molecules like perforin and granzyme B, which can directly target and destroy effector cells or APCs. The second mechanism is the suppression of effector T cells by Treg cells via major histocompatibility complex class II (MHC-II), the primary ligand for the lymphocyte activation gene-3 (LAG-3). The third mechanism involves modulating the activity of indoleamine 2,3-dioxygenase in DCs, reducing tryptophan levels and thereby inhibiting T cell activity. Lastly, Treg cells can inhibit DC function through CTLA-4, binding to which down-regulates CD80 expression, a process enhanced by the CTLA-4 produced by activated Treg cells [19, 20].

Treg cells, a subset of T cells, play a significant role in the immunosuppression of the TME in HNSCC. Their aggregation within the TME is regulated by chemokines and their corresponding receptors, such as CCR4-CCL17/22, CCR8-CCL1, CCR10-CCL28, and CXCR3-CXCL10 [21]. Studies have shown that tumors can expand by converting CD4 + CD25- T cells into Treg cells. The prognostic significance of Treg cells in HNSCC remains a subject of debate in the literature [22]. Elevated levels of Treg cells in circulation or tumor tissue have been associated with poor prognosis, whereas tumor infiltration by Treg cells correlates with improved survival and better local control [23]. Further, it has been observed that Treg cells infiltrating tumors may exert more potent immunosuppressive effects compared to circulating Treg cells [21–23]. This could partly explain the reported inconsistencies regarding Treg cells, which are often identified based on Foxp3 expression. Additionally, biological heterogeneity may contribute to these discrepancies, as Treg cells exhibit characteristics influenced by the location, histology, and molecular profile of the primary tumor [23].

#### Tumor-associated macrophage

**Fig. 5:**
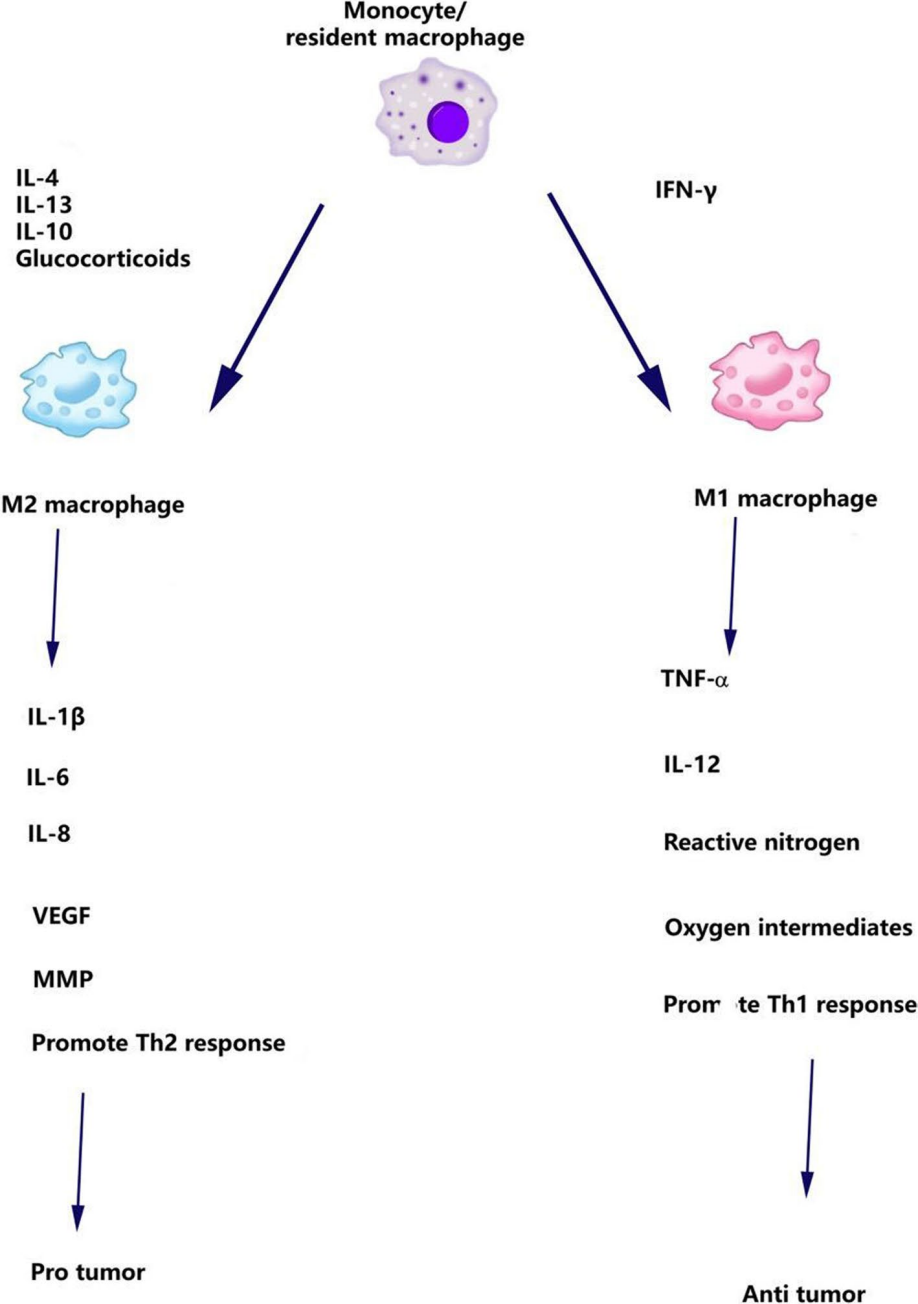
The cytokines produced within the TME can generate macrophages with distinct physiological characteristics. Cytokines such as IL-4 (interleukin 4), IL-13, and IL-10 can induce monocytes to differentiate into tumor-promoting (M2-type) macrophages, while IFN-γ (interferon γ) induces the formation of macrophages with anti-tumor properties (M1-type). M2-type macrophages release molecules such as IL-1β, IL-6, IL-8, VEGFs (vascular endothelial growth factors), and MMPs (matrix metalloproteinases), promoting Th2-type immune responses. On the other hand, M1-type macrophages produce TNF-α, IL-12, reactive nitrogen, and oxygen intermediates, stimulating Th1-type immune responses, and exerting inhibitory effects on tumor formation

Macrophages are tissue-resident monocytes with phagocytic properties, differentiated into two types: M1 and M2, based on their degree of differentiation and functional characteristics. The functional disparity between these two macrophage types is largely influenced by their origin, location, and surrounding environmental factors [24]. M1 macrophages exhibit pro-inflammatory properties and bolster anti-tumor immune responses by producing inflammatory cytokines such as IL-12, IL-23, IFN-γ, and reactive oxygen species. Conversely, M2 macrophages are associated with tumor promotion. They facilitate angiogenesis and contribute to the formation of an immunosuppressive TME by secreting cytokines like IL-10 and TGF-β, thus playing a role in tumor progression and maintenance [25], as depicted in Fig. 5.

TAMs are mature macrophages recruited into the TME, typically exhibiting an M2 phenotype. The recruitment of macrophages into the TME is facilitated by the differentiation of MDSCs either within the bone marrow or directly in the TME, a process regulated by several hematopoietic growth factors including colony-stimulating factor 1 (CSF-1), monocyte chemotactic protein 1 (MCP-1), and various chemokines [23–25]. Hypoxic conditions within the TME further promote TAM migration. Once present in the TME, TAMs significantly contribute to the establishment of an immune-suppressive environment through multiple mechanisms. These include metabolic inhibition of T cells, expression of immune checkpoint molecules like PD-L1, and suppression of natural killer (NK) cell activity [25].

The abundant presence of TAMs within the TME has been identified as an adverse prognostic factor in various cancer types, including breast, gastric, ovarian cancers, and HNSCC. A retrospective study highlighted that in oral squamous cell carcinoma (OSCC), the TME is predominantly composed of M2-phenotype macrophages, characterized by a high percentage of IL-10 and TGF-β [26]. The levels of these cytokines were found to be higher in OSCC compared to normal oral mucosa, with a significantly greater proportion of macrophages in metastatic OSCC than in non-metastatic and control groups. In an experimental HNSCC mouse model, the intratumoral injection of TLR7 and TLR9 agonists, in combination with PD-1 blockade, led to an increased M1/M2 macrophage ratio, enhanced recruitment of CTLs, and subsequent tumor regression [27]. Another study demonstrated that curcumin impedes HNSCC cell migration and invasion by disrupting the feedback loop involving epidermal growth factor and CCL2 production in both macrophages and tumor cells. In light of the role of TAMs in fostering an immunosuppressive TME, strategies targeting TAM recruitment or reversing TAM polarization are increasingly viewed as promising approaches in the treatment of HNSCC [27, 28].

#### Cancer-associated fibroblast

CAFs are a distinct type of fibroblasts characterized by their proliferative and migratory capabilities. They produce various molecules that promote tumor cell proliferation, reshape the ECM, induce tissue stiffening, stimulate tumor progression and metastasis, and influence the tumor immune response [28]. The precise origin of CAFs is not fully understood, but studies by Subramaniam et al. suggest that CAFs may originate from myeloid mesenchymal stem cells or as a result of interactions between tumor cells and fibroblasts [26, 27]. Other proposed origins include cytokine-mediated differentiation of fibroblasts and the malignant transformation of fibroblasts under the influence of hypoxia-induced factor 1α transcription factors. As an integral component of the TME, CAFs play a vital role in tumor angiogenesis, as well as cancer cell invasion and metastasis [26–28]. In the TME, fibroblasts are converted into CAFs via the TGF-β and IL-1β signaling pathways. Functionally, CAFs collaborate with tumor cells to establish an immunosuppressive network, aiding in immune evasion. ECM remodeling mediated by CAFs is a key mechanism contributing to increased tumor interstitial pressure, which facilitates tumor progression and hampers the delivery of therapeutic drugs within the TME. CAFs have been found to inhibit T cell proliferation in HNSCC through VEGF and TGF-β, subsequently inducing immunosuppression by promoting Treg cells aggregation [27, 28]. Additionally, CAFs have been shown to support tumor cell immune evasion by attracting M2 macrophages and MDSCs [29]. Therapeutic strategies targeting CAFs in antitumor treatments typically involve the depletion of CAFs through gene deletion or drug inhibition of cell surface markers. Additionally, these strategies aim to modify CAF activity and function by targeting chemokines, which can alter CAF function. Approaches include activating or deactivating CAFs, targeting CAF-derived ECM, and utilizing CAFs as vehicles for drug delivery. Research in the field of HNSCC regarding CAF-directed therapies is still in its early stages [29].

Immunosuppressive cells hold significant roles within the TME, serving to foster immunosuppression, enable tumor cells to elude immune surveillance, and facilitate tumor progression and metastasis. These cells significantly contribute to the diverse nature of the TME in HNSCC. Addressing their presence poses both challenges and opportunities for therapies. Future studies should prioritize unraveling the interactions among these cells understanding their impact on tumor advancement and immune evasion and developing targeted treatments to regulate their function for better outcomes, in cancer management.

### Antitumor immune cells

#### NK cells

Natural killer (NK) cells, key effector cells of the innate immune system, constitute approximately 10–15% of all peripheral lymphocytes. Resting NK cells are primarily found in the peripheral blood, liver, spleen, and peritoneal cavity. When activated by cytokines, NK cells can migrate and infiltrate tissues that contain pathogen-infected or malignant cells [28, 29]. NK cells are classified into subgroups based on the differential expression of surface markers. Generally defined as CD3-CD56 + cells, they are further subdivided into two groups: CD56dim and CD56bright, based on the density of the CD56 antigen. CD56dim NK cells, which comprise about 90% of total NK cells, are predominantly found in peripheral blood circulation [26–29]. Conversely, CD56bright NK cells, accounting for roughly 10% of the total, are mainly located in secondary lymphoid tissues. CD16 is another commonly used NK cell surface marker, with its expression pattern correlating to CD56 density: CD56dim NK cells typically express high levels of CD16 (CD16high), while CD56bright NK cells are associated with absent or low CD16 expression (CD16-/low) [29, 30].

The primary function of NK cells is to lyse target cells and provide immune regulation. Unlike T cells, NK cells, as direct cytotoxic cells, do not require prior antigen sensitization for their response, enabling them to react rapidly [30]. The activity of NK cells is modulated by a balance between surface inhibitory receptors, such as CD94/NKG2A, KIRs, and T cell immunoglobulin and mucin domain-3 (TIM-3), and activating receptors including CD16 (FcγRIIIa), NKG2D, NKp30, NKp44, NKp46. Under physiological conditions, ligands on the surface of normal cells bind to NK cell inhibitory receptors, transmitting inhibitory signals and maintaining NK cells in a resting state to prevent harm to normal cells [30, 31]. In pathological conditions, such as in tumor cells, a decrease in inhibitory ligands or an increase in activating ligands leads to NK cell activation. Activation occurs through mechanisms like exocytosis of perforin and granzymes, engagement of the Fas ligand (FasL), activation of TNF-related apoptosis-inducing ligand (TRAIL), or induction of antibody-dependent cellular cytotoxicity (ADCC) leading to target cell apoptosis. Additionally, NK cells contribute to immune regulation by secreting cytokines and chemokines, thereby activating the downstream adaptive immune response [29–32].

NK cells play a critical role in the initial defense against tumors, and their reduced numbers, low-level infiltration, or impaired function are common in HNSCC [32]. Studies have found that only low levels of NK cell infiltration are detectable in HNSCC cancer tissues, with a predominance of regulatory CD56 + NK cells over cytotoxic CD56- NK cells [30–33]. Patients exhibiting higher tumor-infiltrating NK cell activity tend to have a better prognosis and a lower incidence of regional and distant lymph node metastases. Conversely, those with low NK cytotoxicity face a higher risk of regional and distant metastases and increased mortality. An immune complex has been identified that inhibits NK cell function in the peripheral blood of these patients [34]. In human papillomavirus (HPV)-positive HNSCC patients, the rate of CD56- NK cell tumor infiltration is higher compared to HPV-negative patients, potentially explaining the better clinical prognosis observed in HPV-positive cases [35]. The presence of the surface antigen CD57, associated with a favorable prognosis and a lower rate of lymph node metastasis, correlates positively with these findings. Peripheral blood analysis reveals that early-stage tumor patients have higher NK cell counts than those with advanced tumors. As with tumor-infiltrated NK cells, the number and cytotoxicity of circulating NK cells decrease in advanced stages, with a higher proportion of CD56 + NK cells compared to CD56- NK cells [34, 35]. Further research indicates that CD56dim NK cells are more prone to spontaneous apoptosis than CD56 + NK cells. Although an increase in the proportion of CD56- NK cells in circulating NK cells has been reported, there is an overall decrease in the number of circulating NK cells and a reduction in their cytotoxicity. This suggests that both the quantity and function of NK cells may be compromised as the tumor progresses [33–36].

Radiotherapy combined with chemotherapy is a common treatment option for locally advanced and metastatic squamous cell carcinoma of the head and neck. A systemic side effect of chemoradiotherapy is the suppression of immune function [36]. One study involving 23 patients undergoing radiotherapy alone observed no significant change in circulating NK cell levels before and after the treatment. However, another study reported a decrease in NK cell numbers following chemotherapy with cisplatin/paclitaxel and carboplatin/docetaxel, while an increase in NK cell numbers was noted after concurrent chemoradiotherapy [37]. The activation level of NK cells in these contexts remains unclear, and hence the impact of NK cells on the efficacy of radiotherapy and chemotherapy warrants further investigation.

NK cells can serve as prognostic and clinicopathological markers in HNSCC. The high expression of the inhibitory antigen, cell adhesion molecule 1 (CEACAM1), has been associated with decreased survival and poor prognosis in HNSCC. CEACAM1 also impedes the signaling of the activating receptor NKG2D, thereby inhibiting the antitumor function of NK cells [35–37]. The receptor-binding cancer antigen expressed on SiSo cells (RCAS1), a ligand for CEACAM1, can induce apoptosis in NK cells, contributing to the immune escape of tumor cells. Studies indicate that high expression of RCAS1 in tumor cells is predictive of a higher tumor grade and increased likelihood of lymph node metastasis [36–38].

In patients with HNSCC, the immune system is often suppressed or inactivated. NK cells play a crucial role in regulating tumor cells through their cytotoxicity and immune regulatory functions. However, tumor cells in HNSCC can evade the regulatory actions of NK cells through various mechanisms. These tumor cells produce a range of cytokines that either enhance the signal transduction of inhibitory receptors, block the signals of activating receptors, or inhibit the recruitment of NK cells via the TME. These actions ultimately lead to a reduction in the anti-tumor functionality of NK cells [31–34].

Inhibition of activating receptors on the surface of NK cells is a common mechanism of immune escape in HNSCC. Programmed death 1 (PD-1) is an activation marker on NK cells, and its high expression is associated with a better overall survival rate [36]. PD-1 positive NK cells, enriched in tumor tissues, lose their activation potential upon binding with programmed death ligand 1 (PD-L1) in the TME [37]. In most cases of HNSCC, the epidermal growth factor receptor (EGFR) is overexpressed, and PD-L1 expression is induced through the EGFR-dependent JAK2/STAT1 pathway. Consequently, HNSCC overexpressing PD-L1 evades NK cell surveillance. Natural Killer Group 2 Member D (NKG2D) is another crucial activating receptor on NK cells, playing a vital role in immune surveillance [34–38]. To escape NK cell surveillance, HNSCC cells secrete various NKG2D ligands (NKG2DLs). High levels of NKG2DLs prevent NK cell infiltration into tumor tissues and reduce their cytotoxicity. However, removing shed NKG2DLs from the plasma of HNSCC patients can restore NK cell function [26, 29]. In recurrent HNSCC, the expression of soluble major histocompatibility complex Class I chain-related peptide A and transforming growth factor-β is elevated, inhibiting NKG2D-dependent NK cell activation. Additionally, reduced expression of active receptors NKp30 and NKp46 has been reported, further contributing to the impaired NK cell function in HNSCC [34, 35].

The overexpression of inhibitory receptors on NK cells and their ligands is another mechanism contributing to the immune escape of HNSCC cells. CD56dim NK cells, in particular, are regulated by Killer Cell Immunoglobulin-like Receptors (KIRs) on their surface [33, 35]. Studies indicate that HNSCC expresses higher levels of KIRs compared to other solid tumors. This suggests that NK cell activity may be inhibited in HNSCC, and patients with this type of cancer might benefit more from therapies targeting KIRs [26, 29, 31]. Research has also identified an increased expression of the inhibitory receptor T cell immunoglobulin and mucin domain-3 (TIM-3) on peripheral NK cells in patients with oral squamous cell carcinoma. The level of TIM-3 expression is significantly correlated with the clinical stage, degree of differentiation, and lymph node metastasis of tumors, indicating its relevance in the pathogenesis and progression of oral squamous cell carcinoma [13, 27, 35].

Researchers have discovered that exosomes isolated from the plasma of patients with HNSCC can reduce the expression level of Natural Killer Group 2 Member D (NKG2D) and inhibit the cytotoxicity of NK cells [28, 31–34]. Notably, NKG2D ligands (NKG2DLs) released through exosomes can down-regulate NKG2D expression more effectively than monomeric NKG2DLs. Other studies have reported that exosomes carrying programmed death ligand 1 (PD-L1) can be isolated from the plasma of HNSCC patients. The level of PD-L1 in these exosomes correlates with tumor activity and lymph node status, suggesting a novel mechanism of tumor immune escape and the potential of exosomes as non-invasive biomarkers for cancer progression and indicators of immune dysfunction [26, 34]. Furthermore, there exists a complex regulatory network between NK cells and other immune effector cells in HNSCC patients, such as DCs, Tregs, and regulatory B cells. This intricate interplay requires further exploration to fully understand the immunological landscape of HNSCC and its implications for treatment strategies [36].

#### Dendritic cells

Dendritic cells (DCs) are pivotal in mediating the body's internal T-cell immune response to cancer. DCs or their precursors can be recruited into the TME, where they differentiate into mature DCs [14–16]. These cells respond to various molecular signals within the TME, including cell death, ineffective activation, and successful maturation. While immature DCs lack the capability to initiate a T-cell response to the tumor and may even induce tolerance, mature DCs can migrate to lymph nodes draining the tumor, initiating T-cell responses. They also recruit T cells into the TME and produce immunostimulatory cytokines, playing a vital role in regulating the TME [13, 18–20]. However, tumors can also suppress the anti-tumor immune response mediated by DCs. The biological mechanisms of this suppression primarily include abnormal expression and function of DCs, which impair their ability to effectively stimulate T cells and orchestrate an effective immune response against the tumor. Understanding and overcoming these mechanisms is critical for enhancing the efficacy of immunotherapies targeting DCs in cancer treatment.

Firstly, abnormal differentiation of dendritic cell (DC) precursors leads to their reduced numbers. The reduction of FMS-like tyrosine kinase 3 ligand (FLT3L) in the TME hampers the terminal differentiation of pre-DCs, while tumor-derived prostaglandins and gangliosides impact both in situ DCs and myelogenesis [19–23]. Secondly, the inhibition of DC maturation results in functional abnormalities. Tumors can directly produce soluble mediators such as IL-10, transforming growth factor β (TGF-β), IL-6, or vascular endothelial growth factor, which interfere with DC activation pathways. Indirectly, tumors can influence DC maturation, for example, by producing colony-stimulating factor 1 to recruit tumor-associated macrophages that inhibit DC maturation [19–23]. Thirdly, tumors can directly affect DC cell activity and function. Tumors can be processed and cross-presented by DCs to TAAs, which promotes the accumulation of partially degraded lipids, interfering with cargo transport within DCs. DC metabolism can be altered by increased accumulation of truncated fatty acids and reduced availability of nutrients and oxygen, impairing DC function. Stimulator of interferon genes (STING) activates DCs through two signaling pathways: chemokines CXCL1/2 and type I interferon (IFN), triggering an anti-tumor immune response [19–23]. Fourthly, phenotypic changes in DCs induce immune tolerance. Tumors can inhibit DC infiltration by reducing the expression of DC chemokines like CC-chemokine ligand 4 (CCL4) or by preventing other cells (e.g., NK cells) from producing chemokines. Tumors also evade detection by limiting the release of activating molecular signals. In such abnormal states, DCs cannot correctly recognize antigens or provide signals for downstream T cell activation [19–23]. Clinical studies have shown that high levels of mature DC infiltration in the tumor immune microenvironment are indicative of a favorable prognosis. DC infiltration reflects the host's immune defense mechanisms and is associated with better prognosis, lower recurrence rates, and fewer metastases [17, 31–35]. This has been observed in malignancies such as lung, breast, colorectal, and gastric cancers. Thus, the involvement of DCs in anti-tumor immunity suggests a direct correlation between DC behavior and disease progression. Understanding the causes of abnormal DC status, their role in health and disease, and developing effective therapies are currently the focus of research [36].

Recent studies have highlighted the importance of different types of DCs in antitumor immunity. In HPV-associated oropharyngeal squamous cell carcinoma (OPSCC) tumors, CD163 + cDC2s have been found to play a significant role in stimulating tumor-infiltrating T cells to exert antitumor effects. OX40 + plasmacytoid DCs (pDCs) in the TME of HNSCC possess a unique immunostimulatory phenotype and cytolytic function. They can collaborate with conventional DCs to generate effective tumor antigen-specific CD8 + T cell responses, thereby promoting antitumor immune function [33–38]. The relationship between DC expression and prognosis in HNSCC has also been a focus of research [36–39]. Studies report that local DC infiltration in head and neck tumor tissue is closely associated with survival rates, tumor recurrence, and metastasis. High DC expression is an important indicator of favorable HNSCC prognosis, and the degree of DC infiltration in tumor tissues with distant metastasis is significantly lower compared to non-metastatic cases [35, 37–39]. Numerous marker proteins target DCs, such as S100, CD1a, CD83, CD207, CD208, CD80, CD11c, CD86, and HLA-DR. CD1a is considered a marker of immature DCs, while CD83 is a marker of mature, activated DCs. The depletion of CD1a + cells around oral squamous cell carcinoma tissues is independently correlated with overall survival and recurrence risk [38–40]. The presence of CD1 + cells near tongue cancer correlates with a better prognosis, lower recurrence rate, and higher survival rate [39]. Tubulin polymerization promoting protein family member 3 (TPPP3) is associated with microtubule dynamics and stability. Its low expression in HNSCC is closely linked to antigen processing and presentation, and the expression of TPPP3 correlates with multiple immune markers in CD8 + T cells and DCs [38–40]. In laryngeal cancer, a higher density of DC infiltration among cancer cells is associated with less local lymph node metastasis and a stronger response of pericancerous lymphocytes, indicating the crucial role of DCs in local antitumor responses [36–41]. Peripheral DCs in HNSCC patients have also been studied. In a study involving cancer patients and controls, it was found that while total peripheral DC levels were similar in both groups, CD11c expression subsets were significantly lower in HNSCC patients, returning to normal levels post-tumor resection [42]. This subtype is crucial for tumor immunity and appears to be suppressed by tumor products. Another study noted a significant reduction in the total number of peripheral blood DCs in HNSCC patients, with the level of reduction correlating with prognosis [43]. Both studies observed an increase in immature DCs with disease progression. Post-tumor resection, the level of immature myeloid-derived DCs decreased, suggesting a direct role of DCs in tumor dynamics [42, 43].

While DCs remain central to the treatment paradigm of HNSCC, significant breakthroughs in leveraging them as a core treatment strategy have yet to be achieved. This may be due to the complex interactions and regulatory effects of various cells and factors within the TME on DCs. Nevertheless, DCs are still considered excellent candidates for therapeutic cancer vaccines. These DC-based vaccines are designed to elicit tumor-specific effector T cells capable of specifically targeting and reducing tumor mass. Additionally, they can induce immune memory, potentially decreasing the risk of cancer recurrence [40–43].

#### CD8 + T cells

CD8 + T cells are critical in protective immunity against intracellular pathogens and tumors. To investigate their role further, researchers conducted a detailed analysis of CD8 + T cells in tumor tissues. They collected tumor tissue samples from patients with kidney cancer and used flow cytometry to analyze the distribution of tumor cells and CD8 + T cells within these tissues. The findings revealed that the percentage of CD8 + T cells in the tumor tissues of all patients ranged from 0.002 to 20%. Notably, patients with a percentage of CD8 + T cells less than 2.2% faced a fourfold higher risk of postoperative disease progression [44, 45].

In scenarios of tumors or chronic infections, CD8 + T cells are subjected to persistent antigen exposure and/or inflammatory signals. This prolonged exposure often leads to a progressive 'exhaustion' of CD8 + T cells’ functions, characterized by a gradual loss of effector functions, increased expression of various inhibitory receptors (such as PD-1 and LAG3), metabolic dysregulation, and diminished memory responses [36, 44]. These functional changes are intricately linked to alterations in transcriptional processes and epigenetic modifications. Additionally, certain molecules present in the TME can also induce CD8 + T cell exhaustion. An early indicator of this exhaustion is a marked decrease in IL-2 secretion, followed by reduced levels of other cytokines like TNF. In this state, T cells may also undergo apoptosis, leading to a significant reduction in the number of virus-specific T cells. These phenomena underscore the complex challenges in managing CD8 + T cell responses in cancer and chronic infectious diseases [44–46].

Antitumor immune cells, pivotal in the immunological counteraction against tumors, often encounter efficacy attenuation due to the immunosuppressive milieu established by the TME. Prospective research endeavors should prioritize strategies to surmount the immunosuppressive hurdles presented by the TME, augmenting both the infiltration and functional capacity of these immune effectors. Delving into innovative immunotherapeutic approaches and the enhancement of extant treatment modalities promises to yield more efficacious and tailored therapeutic interventions for patients afflicted with HNSCC.

### Effect and component of ECM

#### Tumor promoting effect of ECM

Extensive remodeling of ECM results in heightened collagen density and alterations in tissue rigidity, both of which are intricately linked to the malignant characteristics of tumors. Altered ECM impedes drug access to tumor cells and facilitates tumor cell proliferation and migration by inducing epithelial-mesenchymal transition and angiogenesis [47]. Additionally, the ECM plays a pivotal role in modulating tumor-associated immunosuppression by influencing the proliferation, distribution, and function of myeloid cells.

#### Antitumor effects of ECM

Typically, drug distribution within the tumor matrix primarily relies on diffusion. Studies have demonstrated that solid tumor tissues often exhibit elevated collagen density and increased tissue stiffness, primarily attributed to the accumulation of ECM fibers [48]. While the excessive deposition of collagen fibers in the tumor ECM serves as an effective barrier to impede tumor cell dissemination, it also hinders the penetration of chemotherapy drugs into tumor cells. Consequently, the normalization of ECM should be a critical consideration during chemotherapy to enhance drug delivery [11, 47, 48].

#### Tumor promoting cytokines

In the majority of HNSCC cases, EGFR has been demonstrated to upregulate immature DCs in the TME, leading to impaired T cell function. IL-10, produced by Th2 cells, can induce the recruitment of M2 macrophages and increase the number of Treg cells, thereby suppressing DC function [49].

#### Antitumor cytokines

Type I IFNs can induce the expression of MHC Class I molecules in tumor cells, promote DC maturation, and enhance anti-tumor immunity. IL-12 and IL-18 can stimulate Th1 cell immune responses and initiate anti-tumor immunity [23, 37–39]. Additionally, CXCL9, CXCL10, TNF-α, IL-1, IL-6, and IL-12 induce the M1 phenotype in TAMs. However, some cytokines possess dual pro-tumor and anti-tumor properties, such as IL-6. Functionally, IL-6 inhibits DC maturation, thereby suppressing the activation of neutrophils, macrophages, NK cells, and T cells, which is closely associated with the prognosis of HNSCC. IL-6 can also induce the production of M1 macrophages to generate an anti-tumor immune response [46, 47].

In summary, the ECM plays a dual role in HNSCC. It contributes to tumor growth via structural remodeling and immunosuppressive modulation, but it also possesses inherent anti-cancer properties through physical containment of neoplastic cells and immunological interaction. Future research needs to focus on understanding the interplay between the ECM and the TME with a particular emphasis on developing advanced therapeutic interventions. These interventions should strategically reduce the cancer promoting effects of the ECM, making it a crucial target for effective management of HNSCC.

## HNSCC-related immunotherapy

### PD-1/PD-L1 and its inhibitory antibody

PD-1 is an immune checkpoint receptor protein expressed on the surface of activated T cell membranes. It binds to two ligands, PD-L1 and PD-L2, to reduce effector T cell activity and halt immune responses, thereby preventing excessive inflammation or autoimmune reactions [35–37]. PD-L1 and PD-L2 belong to the B7 superfamily of proteins, and their expressions are increased in various tumors, including HNSCC. PD-L2 is mainly expressed in antigen-presenting cells but can be induced to express on the surface of tumor cells under the influence of inflammatory cytokines. Several studies have shown significant remissions in patients with metastatic melanoma and non-small cell lung cancer after 12 and 16 cycles of treatment with anti-PD-1/PD-L1 antibodies, respectively [41–43].

Pembrolizumab is a highly specific anti-PD-1 monoclonal antibody that disrupts PD-1 interactions with its ligands, PD-L1 and PD-L2, effectively relieving PD-1-induced immune suppression [56]. In the Phase Ib trial (KEYNOTE-012), 60 patients with PD-L1-positive, relapsed, or metastatic squamous cell carcinoma of the head and neck were administered Pembrolizumab every two weeks for 24 months or until disease progression, inability to tolerate adverse reactions, or other conditions preventing treatment. The trial reported an overall response rate of 18%, with progression-free survival and overall survival of 2 and 13 months, respectively [57, 58]. Subsequently, a larger Phase I clinical trial was conducted, including 132 participants irrespective of PD-L1 status. In this trial, the overall response rate remained at 18%, with progression-free survival of 2 months and overall survival of 8 months. Importantly, PD-L1 positive patients showed a significantly higher response rate compared to PD-L1 negative patients [43, 47]. Based on these promising results, the FDA approved Pembrolizumab in 2016 for the treatment of platinum-resistant relapsed or metastatic HNSCC [51]. Pembrolizumab is currently being investigated in Phase III trials for HNSCC to assess its efficacy and safety as a monotherapy or in combination with standard first-line chemotherapy [54].

Nivolumab, an anti-PD-1 antibody, has exhibited promising efficacy in the treatment of HNSCC, particularly in cases resistant to platinum-based therapies [53–55]. Its recent FDA approval was based on compelling evidence from a Phase III clinical trial, wherein it demonstrated a notable improvement in median survival rates. Specifically, patients treated with Nivolumab exhibited a median survival of 7.5 months compared to 5.1 months with standard therapies [58]. This pivotal finding signifies the potential of Nivolumab as a superior therapeutic option for advanced HNSCC, offering renewed optimism for patients with limited treatment response rates.

Durvalumab is a highly specific and affinity-driven PD-L1 inhibitory antibody capable of obstructing the binding of PD-L1 to its receptors PD-1 or CD80. Importantly, it does not interfere with the activity of PD-L2. In a Durvalumab I/II trial involving patients with HNSCC, the overall response rate was 12%, with a notable 25% response rate observed in patients who were PD-L1 positive [55–57]. Furthermore, the disease response rate at 24 weeks reached 16% (25% in PD-L1 positive patients), and adverse reactions were reported in only 7% of patients, with no treatment-related deaths recorded. Currently, Durvalumab is progressing through Phase III clinical trials, evaluating various monotherapy and combination therapy approaches in the treatment of HNSCC [58, 59].

### CTLA-4 and its inhibitory antibody

CTLA-4 plays a crucial role in maintaining normal immune balance, but tumor cells exploit its negative immune regulation to inhibit T cell activation. CTLA-4 inhibitors are important ICIs in cancer therapy [23–37, 60]. In the TME of HNSCC, Treg cells regulate the expression of CTLA-4 on their cell surface to suppress anti-tumor immunity. Therefore, CTLA-4 inhibitors effectively reverse the immunosuppression induced by Treg cells. The mechanism of blocking the PD-1 signaling pathway by PD-1/PD-L1 inhibitors is considered immune normalization therapy [56, 57]. In contrast to the well-known immunosuppressive mechanism of PD-1/PD-L1, CTLA-4 inhibitors often enhance immune responses, leading to improved immune cell-mediated killing of cancer cells. However, this enhancement is often accompanied by more toxic side effects [58, 59].

Ipilimumab, a CTLA-4 inhibitor, was approved by the U.S. Food and Drug Administration (FDA) on March 25, 2011, for the treatment of unresectable advanced melanoma. It was later approved as an adjuvant therapy for stage III melanoma in October 2015 [43, 44]. Ipilimumab works by blocking the binding of CTLA-4 to the B7 ligand, ensuring the activation and proliferation of T cells, ultimately leading to its anti-tumor effect [32]. Although Ipilimumab demonstrated better overall survival in clinical trials and significantly improved clinical efficacy compared to the control group, it commonly causes toxic side effects, including rash, diarrhea, fatigue, itching, headache, weight loss, and nausea during clinical use. Ipilimumab can also induce autoimmune diseases affecting the digestive system, skin, nervous system, and hormone-producing glands [16, 19–23]. These side effects are mainly due to the specificity of CTLA-4 targeting, which leads to immune enhancement and the induction of clinical toxicities. Due to the potentially life-threatening immune-mediated side effects associated with Ipilimumab, its approved label includes a black box warning to inform patients about these potential side effects [25, 32–34].

### Immunotherapy combined with chemotherapy

Cytotoxic chemotherapy has traditionally been viewed as immunosuppressive due to its myelosuppressive effects. However, recent evidence indicates that chemotherapy can also act as an immune stimulant through two primary mechanisms: First, it enhances the immunogenicity and T cell infiltration of CD8 + effector T cells, which induces high expression of PD-L1 in tumor cells and promotes the presentation of TAAs on MHC Class I molecules. Second, it contributes to the elimination of immunosuppressive cells such as Treg, MDSCs, and TAMs [31–34, 56].

The KEYNOTE-407 trial, a double-blind, randomized, controlled Phase III study, demonstrated that the combination of pembrolizumab and chemotherapy resulted in a longer overall survival compared to placebo with chemotherapy (15.9 vs. 11.3 months) [48]. Similarly, the KEYNOTE-048 trial, a randomized Phase III study, compared pembrolizumab alone or in combination with chemotherapy (cisplatin or carboplatin with 5-FU) against the EXTREME regimen (Erbitux with platinum-based chemotherapy and 5-FU) in patients with R/MHNSCC. It was observed that while the overall response rate (ORR) for the pembrolizumab monotherapy arm was lower than the chemotherapy arm, pembrolizumab demonstrated a significantly longer OS in patients with a combined positive score (CPS) of 1 or more, compared to the chemotherapy arm [63]. Additionally, pembrolizumab monotherapy was found to be as effective as the EXTREME regimen in the general population, irrespective of CPS [65]. In cases with CPS ≥ 20, CPS ≥ 1, and in the general population, the combination of pembrolizumab and chemotherapy showed superior OS. Based on these efficacy and safety findings, pembrolizumab, alone or in combination with chemotherapy, has been approved as a first-line treatment for R/MHNSCC [35–37, 67].

### Immunotherapy combined with radiation therapy

Current data suggest that the combination of radiotherapy and ICIs may transform 'cold' (non-immunogenic) tumors into 'hot' (immunogenic) tumors. This is evidenced by the up-regulation of PD-L1 expression in tumor cells following radiotherapy [66–68]. Furthermore, local radiotherapy appears to exert effects on distant, non-irradiated metastatic lesions. This observation implies that local radiation can activate an immune response by facilitating the release of TAAs and enhancing antigen presentation. It may also be involved in upregulating the MHC Class I complex in tumor cells and reducing the number of Treg cells [69, 70].

In a Phase II randomized clinical trial, the combination of targeted single focal radiation with Opdivo (nivolumab) was deemed safe; however, no significant differences were observed in overall response rate (ORR), progression-free survival (PFS), or overall survival between the treatments [71]. Consequently, the authors concluded that Opdivo combined with stereotactic radiotherapy does not induce the abscopal effect. In a separate Phase I/II trial, durvalumab combined with radiotherapy was administered to patients unsuitable for surgery or with metastatic tumors that had at least 5% of PD-L1-positive cells stained at multiple disease sites [71–73]. This combination achieved an ORR of 60%, with adverse reactions reported as transient and manageable. These findings suggest the safety of ICIs in combination with radiotherapy [72, 73]. However, evidence of a synergistic effect between ICI and radiotherapy still requires validation in randomized clinical studies. Currently, over 40 clinical trials ranging from Phase I to III are underway to evaluate the effects of combining radiotherapy with ICI in head and neck cancer [45, 48–52]. Several Phase I/II clinical trials assessing Opdivo or pembrolizumab combined with chemoradiotherapy (CRT) have established the safety and feasibility of this approach [67, 69–71]. Two ongoing Phase III clinical trials, NIVOSTOP and KEYNOTE-412, are investigating the potential synergistic effects of Opdivo and pembrolizumab with cisplatin-based CRT in patients with locally advanced HNSCC [38, 52]. In preclinical studies, radiation and chemotherapy have been observed to up-regulate the expression of immune checkpoints in tumor-associated lymphocytes, including PD-1, TIM-3, and CTLA-4. Additionally, an increase in both CD4 + and CD8 + T cells was noted in response to treatment with radiation and chemotherapy [63–65].

### Immunotherapy combined with viral therapy

Growing evidence indicates that the mechanisms by which oncolytic viruses kill tumor cells include direct lysis, induction of innate immunity, stimulation of adaptive immune responses, disruption of the tumor vascular system, and modification of the tumor-suppressive microenvironment. Talimogene laherparepvec (T-VEC), a modified herpes simplex virus type I, has been genetically engineered by deleting the ICP34.5 and ICP47 genes and inserting a sequence encoding human granulocyte–macrophage colony-stimulating factor [38, 41, 52, 57]. In a Phase II trial involving 50 patients with unresectable melanoma treated with T-VEC and pembrolizumab, an overall response rate (ORR) of 26% was observed [61]. Among patients responding to the combination therapy, increases were noted in CD8 + T cell counts, PD-L1 protein expression, and IFN-γ gene expression. Importantly, no new or dose-limiting side effects were reported in 21 patients, aligning with the known side effects of T-VEC or pembrolizumab when used individually [58–64]. Oncolytic virus therapy has the potential to enhance the efficacy of the PD-1 inhibitor pembrolizumab by altering TME. In a phase I trial investigating the combination of T-VEC and pembrolizumab in R/MHNSCC, it is hypothesized that this combination could enhance the antitumor activity of effector T cells [65, 78, 79].

### PD-1/PD-L1 inhibitor combined with CTLA-4 inhibitor therapy

The immune escape mechanisms of tumors are complex, and the efficacy of PD-1/PD-L1 inhibitors alone is limited. However, combining ICIs with other ICI inhibitors may yield synergistic antitumor effects. Clinical trials exploring these combinations are currently underway in HNSCC [60–62, 73]. The PD-1 and CTLA-4 pathways have complementary but distinct mechanisms of action. The PD-1 pathway predominantly functions during the response phase in the TME, whereas the CTLA-4 pathway is mainly active in the lymph nodes during the initiation phase of the immune response, facilitating the proliferation of effector T cells and reducing Treg-mediated inhibition of T cell responses [74, 75]. PD-1 modulates T cells by binding to the ligand CD80, which also interacts with CTLA-4. Immunotherapies targeting the combined inhibition of CTLA-4 and PD-1 have demonstrated efficacy in melanoma, renal cell carcinoma, and non-small cell lung cancer [56, 73–76].

In the CONDOR trial, a randomized, open-label, multicenter Phase II clinical study, 267 patients with PD-L1 negative disease who had progressed after platinum-based therapy in a recurrent/metastatic (R/M) setting were divided into three treatment groups: Durvalumab (D), Tremelimumab (T), and a combination of Durvalumab and Tremelimumab (D + T). The trial found no significant differences in efficacy endpoints among these three treatment modalities [65–67, 77]. A more diverse cohort of patients, both PD-L1 positive and negative, was included in a subsequent randomized Phase III clinical trial [54, 58–61]. The results of this trial indicated that neither the D + T nor the D group demonstrated improved overall survival compared to the standard-of-care (SOC) chemotherapy group, with a median OS of 7.6 months in the D group, 6.5 months in the D + T group, and 8.3 months in the SOC group. Overall response rates (ORRs) were also similar across the three groups. However, the duration of response to the combination immunotherapy regimen was more sustained than in the SOC group, with 7.4 months in the D + T group, 2.9 months in the D group, and 3.7 months in the chemotherapy group [62, 63]. Previous studies combining anti-PD-1 and anti-CTLA-4 therapies have not demonstrated the superiority of dual immune checkpoint blockade in patients with R/MHNSCC who progressed after platinum-based chemotherapy [58, 64–67]. Currently, two large Phase III trials, CheckMate 651 and KESTREL, are underway to evaluate the efficacy of dual immune checkpoint blockade in patients with previously untreated R/M HNSCC.

The current landscape of HNSCC-related immunotherapy, marked by the use of PD-1/PD-L1 and CTLA-4 inhibitors, has shown promising results, particularly in patients resistant to conventional therapies. These approaches, however, are not universally effective and are often associated with specific challenges, including the development of resistance and variable patient responses. The effectiveness of these therapies is influenced by factors such as PD-L1 expression, tumor mutational burden (TMB), and interferon signatures. Future research should focus on enhancing the efficacy and broadening the applicability of immunotherapies in HNSCC. This includes exploring combinations of ICIs with traditional therapies like chemotherapy and radiotherapy, and investigating novel agents like oncolytic viruses. Developing predictive biomarkers to identify responders and tailoring therapies to the unique TME are essential steps towards more personalized and effective treatment strategies for HNSCC patients.

## Factors influencing the effectiveness of immunotherapy

Clinical trials involving patients with R/MHNSCC receiving monotherapy with PD-1/L1 inhibitors have yielded mixed results. Influencing factors identified include PD-L1 expression, HPV status, TMB, and interferon response [78].

### The expression of PD-L1

The presence of PD-L1 expression on immune cells in tumor biopsies indicates a pre-existing anti-tumor adaptive immune response, which is associated with an enhanced therapeutic effect. Current studies in HNSCC suggest that the Combined Positive Score (CPS)—the number of PD-L1 positive cells, including tumor cells, lymphocytes, and macrophages, relative to total tumor cells—is a more effective measure compared to the Tumor Proportion Score (TPS), which only evaluates PD-L1 expression on tumor cells [76–79]. The superiority of CPS over TPS in predicting clinical response to HNSCC immunotherapy was first reported in the Phase III KEYNOTE-048 trial. This trial was a prospective 1:1:1 randomized study of 882 patients assigned to either the pembrolizumab group, the pembrolizumab plus chemotherapy group, or the EXTREME regimen group with cetuximab plus chemotherapy [57, 80]. At the second interim analysis, pembrolizumab significantly improved overall survival compared to cetuximab (median 14.9 vs. 10.7 months, P = 0.0007) for patients with CPS ≥ 1 (12.3 vs. 10.3 months, P = 0.00086), and was not inferior to cetuximab in the total patient population (11 and 10.7 months, respectively) [54, 81]. Overall, KEYNOTE-048 demonstrated that pembrolizumab surpassed the standard of care for patients with CPS ≥ 20 and CPS ≥ 1. Consequently, in June 2019, the FDA approved pembrolizumab as a first-line treatment for incurable R/MHNSCC based on this study [81]. However, the KEYNOTE-055 trial revealed that while PD-L1 positive patients exhibited the anticipated higher response rate, PD-L1 negative patients also showed significant therapeutic benefit, indicating the need for further research into the relationship between PD-L1 expression levels and therapeutic efficacy [82]. In summary, PD-L1 expression on immune cells is a key indicator of a pre-existing anti-tumor adaptive immune response, and its presence is associated with enhanced therapeutic effects in HNSCC.

### TMB

Multiple studies have established TMB as a promising biomarker for predicting responses to immunotherapy. An increased TMB has been linked to enhanced responses to PD-1 inhibition and prolonged PFS in non-small cell lung cancer (NSCLC) [32, 46, 83]. In the context of HNSCC, TMB was evaluated using a cutoff of ≥ 102 exon mutations in the KEYNOTE-012 study and was found to be positively correlated with response to immunotherapy. Supplementary data from 126 patients undergoing anti-PD-1/L1 treatment indicated that responders typically had a high TMB, suggesting that TMB may be a critical factor influencing the efficacy of ICIs [82, 83]. In brief, a higher TMB is associated with improved responses to PD-1 inhibition therapies, reflecting the enhanced visibility of tumor cells to the immune system. However, the variability in TMB across patients and its correlation with treatment outcomes underscores the need for more research in this area.

### Interferon

Interferon (IFN) is recognized for its role in the spontaneous recruitment of cytotoxic T cells triggered by tumor-induced innate immunity, a critical process in establishing an inflammatory TME [84]. This is particularly relevant in HNSCC, where the relationship between interferon and ICI treatment was investigated in the KEYNOTE-012 trial [57, 84]. In pre-treatment biopsies, six IFN-γ-related genes (IDO1, CXCL10, CXCL9, HLADRA, STAT1, IFN-γ) were assessed [62, 63]. The findings indicated that an IFN-γ gene signature was associated with better overall responses. Both best overall response (BOR) and PFS were statistically significant, suggesting that this gene signature may serve as a potential biomarker for predicting resistance to immunotherapy due to its high negative predictive value [85]. In conclusion, interferon signatures play a crucial role in modulating the immune response against HNSCC, significantly influencing the success of immunotherapy treatments. The presence of specific interferon-related genes correlates with improved patient response to immunotherapies, highlighting their potential as predictive biomarkers.

## Outlook

Immunotherapy has demonstrated positive therapeutic effects in some patients with HNSCC. However, in many cases, TME can induce drug resistance through compensatory feedback mechanisms and dynamic evolution, which may impede the effectiveness of immunotherapy and potentially lead to tumor hyperprogression. Given the dynamic and inhibitory nature of the TME, further research is needed to explore immunotherapeutic targets involving molecules or signaling pathways within the TME. The main factors limiting the efficacy of immunotherapy in HNSCC include a low response of the host immune system to TAAs, poor immune cell infiltration in the tumor, and the development of an immunosuppressive TME. Considering these challenges, there is a need for identifying predictive biomarkers sensitive to various immunotherapies. Unlike prognostic biomarkers, the field of predictive biomarkers is less developed. Effective predictive biomarkers could forecast the success of specific immunotherapies, leading to substantial improvements in treatment outcomes and enhancing our understanding of the interactions between tumor cells and the TME. Investigating the potential of combining ICIs with other therapeutic strategies is crucial. Optimizing combinations, such as integrating traditional cancer treatments like radiotherapy or chemotherapy with immunotherapy, or combining multiple immunotherapies, could significantly enhance the overall effectiveness in eradicating tumor cells. Additionally, novel immune activation strategies like oncolytic viruses could modify the local immune state of the TME, promoting an inflammatory immune microenvironment conducive to antitumor activity.

## Data Availability

Not applicable.

## Abbreviations

HNSCC: Head and neck squamous cell carcinoma
R/MHNSCC: Recurrent or metastatic head and neck squamous cell carcinoma
TME: Tumor microenvironment
ICI: Immune checkpoint inhibitor
OS: Overall survival
CTL: Cytotoxic T lymphocyte
TAAs: Tumor-Associated antigens
APCs: Antigen presenting cells
DCs: Dendritic cells
TCR: T cell receptor
MHC-I/II: Major histocompatibility complex I/II
PD-1: Programmed death receptor-1
CTLA-4: Cytotoxic T-lymphocyt-associated protein-4
PD-L1/2: PD-1 ligand 1/2
MDSC: Myeloid-derived suppressor cells
TAM: Tumor-associated macrophage
CAF: Cancer-associated fibroblast
ECM: Extracellular matrix
TIM-3: T cell immunoglobulin and mucin domain-3
TRAIL: TNF-related apoptosisinducing ligand
HPV: Human papilloma virus
CEACAM1: Cell adhesion molecule 1
RCAS1: Receptor binding cancer antigen expressed on SiSo cells
NKG2D: Natural Killer Group 2 Member D
NKG2DLs: Natural Killer Group 2 Member D ligands
KIRs: Killer Cell Immunoglobulin-like Receptors
FLT3L: FMS-like tyrosine kinase 3 ligand
CCL4: CC-chemokine ligand 4
OPSCC: Oropharyngeal squamous cell carcinoma
TPPP3: Tubulin polymerization promoted protein family member 3
ORR: Overall Response Rate
CPS: Combined positive score
PFS: Progression-Free Survival
SOC: Standard chemotherapy regimen
TPS: Tumor proportion scores
